# Gut microbiome and cardiometabolic comorbidities in people living with HIV

**DOI:** 10.1186/s40168-024-01815-y

**Published:** 2024-06-14

**Authors:** Marius Trøseid, Susanne Dam Nielsen, Ivan Vujkovic-Cvijin

**Affiliations:** 1 Research Institute of Internal Medicine, Oslo University Hospital Rikshospitalet, Oslo, Norway; 2 Section for Clinical Immunology and Infectious Diseases, Oslo University Hospital Rikshospitalet, Oslo, Norway; 3 Institute of Clinical Medicine, University of Oslo, Oslo, Norway; 4 Department of Infectious Diseases, Rigshospitalet, University of Copenhagen, Copenhagen, Denmark; 5 Department of Clinical Medicine, University of Copenhagen, Blegdamsvej 3B, Copenhagen, 2200 Denmark; 6 Department of Surgical Gastroenterology and Transplantation, Rigshospitalet, University of Copenhagen, Blegdamsvej 9, Copenhagen Oe, 2100 Denmark; 7 Department of Biomedical Sciences, Cedars-Sinai Medical Center, Los Angeles, CA USA; 8 Karsh Division of Gastroenterology & Hepatology, Department of Medicine, Cedars-Sinai Medical Center, Los Angeles, CA USA; 9 F. Widjaja Inflammatory Bowel Disease Institute, Cedars-Sinai Medical Center, Los Angeles, CA USA; 10 Samuel Oschin Comprehensive Cancer Institute, Cedars-Sinai Medical Center, Los Angeles, CA USA

## Abstract

**Background:**

Despite modern antiretroviral therapy (ART), people living with HIV (PLWH) have increased relative risk of inflammatory-driven comorbidities, including cardiovascular disease (CVD). The gut microbiome could be one of several driving factors, along with traditional risk factors and HIV-related risk factors such as coinfections, ART toxicity, and past immunodeficiency.

**Results:**

PLWH have an altered gut microbiome, even after adjustment for known confounding factors including sexual preference. The HIV-related microbiome has been associated with cardiometabolic comorbidities, and shares features with CVD-related microbiota profiles, in particular reduced capacity for short-chain fatty acid (SCFA) generation. Substantial inter-individual variation has so far been an obstacle for applying microbiota profiles for risk stratification. This review covers updated knowledge and recent advances in our understanding of the gut microbiome and comorbidities in PLWH, with specific focus on cardiometabolic comorbidities and inflammation. It covers a comprehensive overview of HIV-related and comorbidity-related dysbiosis, microbial translocation, and microbiota-derived metabolites. It also contains recent data from studies in PLWH on circulating metabolites related to comorbidities and underlying gut microbiota alterations, including circulating levels of the SCFA propionate, the histidine-analogue imidazole propionate, and the protective metabolite indole-3-propionic acid.

**Conclusions:**

Despite recent advances, the gut microbiome and related metabolites are not yet established as biomarkers or therapeutic targets. The review gives directions for future research needed to advance the field into clinical practice, including promises and pitfalls for precision medicine.

Video Abstract

**Supplementary Information:**

The online version contains supplementary material available at 10.1186/s40168-024-01815-y.

## Introduction

Although modern antiretroviral therapy (ART) reduces detectable HIV virus levels to a minimum in people living with HIV (PLWH), a higher morbidity and a shorter life expectancy remain [1, 2]. In particular, PLWH have increased relative risk of inflammatory-driven comorbidities including cardiovascular disease, cancer, kidney, liver, bone, and neurocognitive disease [3].

From 2015, WHO has recommended treatment of all PLWH. Recent data from the Antiretroviral Therapy Cohort Collaboration (ATCC) showed improvements in life expectancy for PLWH that started ART from 2015 and onwards compared to those PLWH that started ART 1999–2014. However, despite these improvements, some groups of PLWH, most notably women living with HIV and PLWH with lower CD4 T cell counts, still have not achieved life expectancy comparable to that in the background population, despite suppressed viral load and no prior AIDS [4]. It is well established that PLWH have a higher burden of comorbidity, and comorbidities occur at a younger age in PLWH. [5] This was elegantly shown in the Dutch AGEhIV study where PLWH were compared to population controls matched on lifestyle including sexual behavior [6]. Importantly, this cohort was followed prospectively for 5.9 years, and number of comorbidities at baseline was associated with an increased risk of death (hazard ratio 3:33 per additional comorbidity) [7] indicating that comorbidities are likely to contribute to excess mortality in PLWH. Likewise, in a study from the Danish HIV Cohort, the probability of survival was dramatically reduced in PLWH with comorbidites [8]. Importantly, despite universal rollout of ART from 2015, PLWH still have fewer years without comorbidity than controls from the general population [9]. Worldwide populations of PLWH are aging, and a recent study from the United States estimated that 23% of ART users will be aged ≥ 65 years in 2030 [10]. Since the incidence of comorbidities increases with increasing age, the absolute burden of comorbidities in PLWH is likely to increase.

Cardiovascular disease (CVD) and diabetes both rank among the top 10 causes of disability-adjusted life years (DALYs), while HIV ranks 11 [11]. Hence, any adverse interaction between HIV and these diseases is likely to have a major impact on health in PLWH, and PLWH do seem to be disproportionately affected by comorbidities. CVD is probably the most well-studied comorbidity in PLWH, and in a recent systematic review across 80 studies that included nearly 800,000 PLWH and a total follow-up of 3.5 million person-years, the crude rate of CVD was 61.8 per 10,000 person-years. Importantly, in comparison with persons without HIV, the risk ratio for cardiovascular disease was just above two. Given the increased risk of ischemic CVD [12], it is not surprising that PLWH also have increased risk of heart failure [13] (HF) with the highest risk among PLWH with lower CD4 T-cell counts or ongoing viral replication [14, 15]. PLWH also have high prevalence of electrocardiographic alterations and seem to be at higher risk of sudden cardiac death [16–19]. Other manifestations of CVD that may be more prevalent among PLWH include aortic aneurysms and peripheral artery diseases [12, 20–24], although these findings are not entirely consistent. Across several studies and different manifestations of CVD, lower CD4 T-cell counts and/or ongoing viral replication is associated with higher risk.

The main risk factor for CVD in both the general population and in PLWH is smoking. Unfortunately, PWLH are more likely to smoke than persons without HIV [25], and smoking is associated with higher risk of myocardial infarction in PLWH than in the general population [26]. Another important risk factor for CVD in PLWH is inflammation, including elevated levels of interleukin (IL)-1 and IL-6 [27, 28]. The Canakinumab Anti-Inflammatory Thrombosis Outcome Study (CANTOS) found that anti-inflammatory therapy with canakinumab, a monoclonal antibody blocking IL-1β, led to a lower rate of CVD than placebo [29], providing evidence to the role of inflammation in the pathogenesis leading to CVD. Chronic inflammation and immune activation are hallmarks of HIV infection, and even well-treated PLWH have higher levels of inflammation and immune activation [30], which is associated with higher CVD risk [31, 32].

Immune activation and inflammation, in turn, may be driven by a number of factors including lifestyle as indicated by a study from the Comorbidity in Relation to AIDS (COBRA) cohort [33]. Furthermore, the prevalence of obesity is increasing in PLWH, and several inflammatory pathways are shared between obesity and treated HIV infection [34]. In the Copenhagen Comorbidity in HIV (COCOMO) study, it was found that abdominal obesity is more common in PLWH than in the general population [35], and abdominal obesity was closely linked to inflammation [36]. Unsurprisingly, the prevalence of metabolic syndrome and diabetes in PLWH is high [37, 38], and a recent meta-analysis found the pooled incidence rate of overt diabetes to be 13.7 per 1000 person-years of follow-up. At present, it is still debated if HIV is an independent risk factor for diabetes [38], but HIV and diabetes are both associated with increased inflammation as manifest by increased levels of proinflammatory markers and monocyte activity as well as an increased risk of CVD.

As such, cardiometabolic comorbidities are common in PLWH and associated with inflammation. Identifying modifiable risk factors is therefore of utmost biomedical importance. The microbiome could be one of several driving factors, along with viral replication, ART toxicity, lipodystrophy, traditional risk factors, coinfections, and past and present immunodeficiency [3]. This review will cover updated knowledge and recent advances in our understanding of the gut microbiome and comorbidities in PLWH, with specific focus on cardiometabolic comorbidities and inflammation. It also contains recent data on circulating metabolites related to comorbidities and underlying gut microbiota alterations.

## The gut microbiota and disease-related dysbiosis

### Gut microbiota alterations in PLWH: confounders and context

The advent of 16S rRNA-based microbiome characterization enabled comprehensive investigations of associations between gut microbiome composition and features of HIV infection. After several early studies investigating the microbiome in PWH had been completed, it was discovered that sexual behavior has a significant impact on gut microbiome composition including, most notably, an increased abundance of *Prevotella* in men who have sex with men (MSM) [39, 40]. As MSM comprise the predominant population of PLWH in many sites in Western Europe and the United States, and as the general population is predominantly non-MSM, comparison of random samplings of PLWH and random samplings of the general population is prone to be confounded by sexual behavior. Indeed, several early HIV microbiome studies were not intentionally matched for sexual behavior, and these studies uniquely reported an increased abundance of *Prevotella* in PLWH [41]. Studies in which PLWH and population controls were matched for sexual behavior have not consistently found enrichment of *Prevotella* in PLWH [42–45]. Within MSM, this taxon was enriched in those who took part in recent anal receptive intercourse as compared to those that did not [46], suggesting *Prevotella* is linked with sexual behavior. As sexual behavior has been shown to have a dominant impact on the microbiome that is greater than HIV serostatus itself [39, 46, 47], microbiome studies addressing hypotheses related to HIV face challenges in circumventing the role of sexual behavior in driving microbiome composition patterns.

**Fig. 1:**
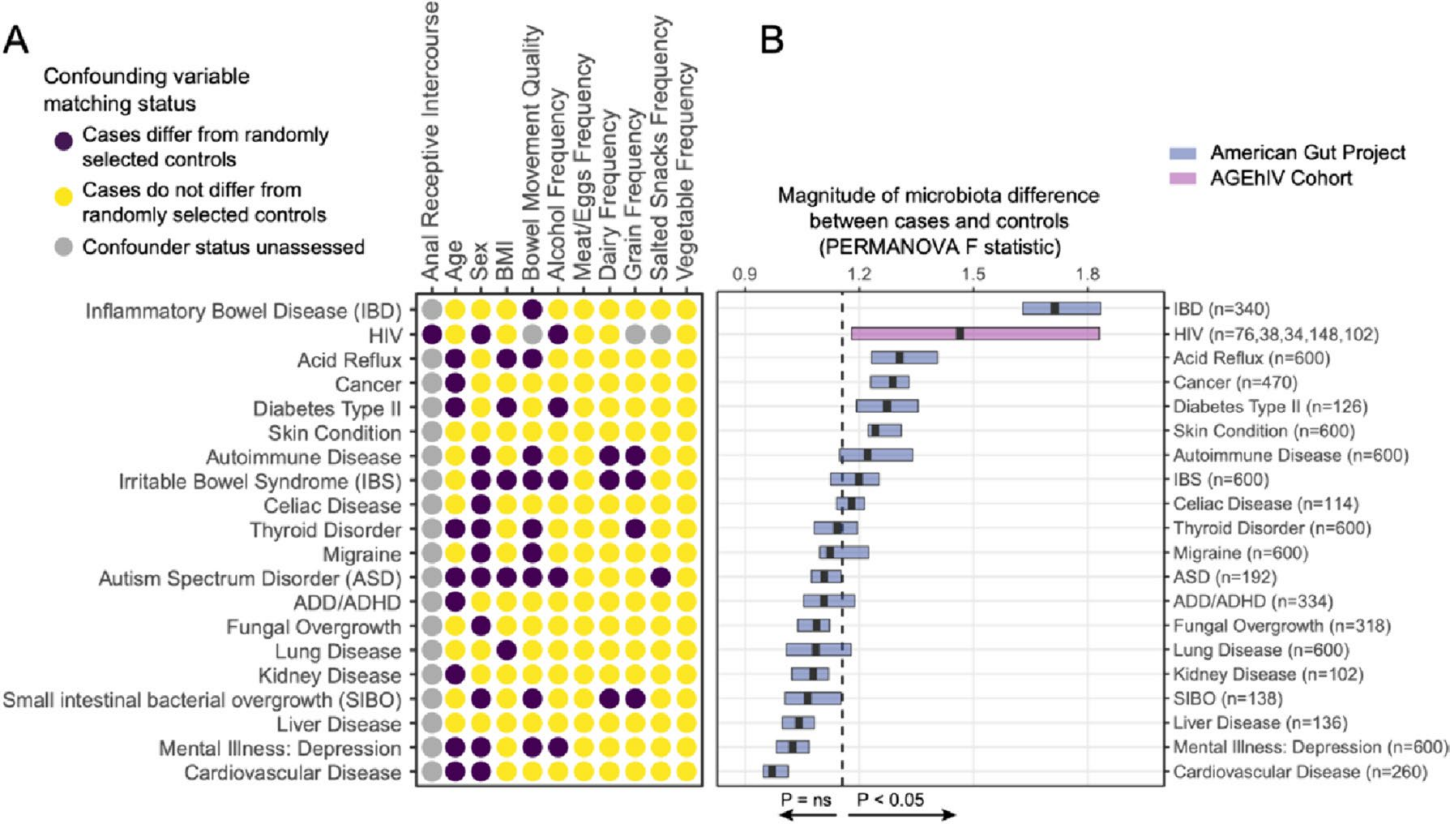
Gut microbiota differences in cases versus unaffected controls across 20 common human diseases. **A** For diseases assessed using the American Gut Project, differences between cases and controls in distribution of confounding variables were assessed as previously described by comparing cases to randomly selected controls [48]. For PLWH, previously reported differences between PLWH and the HIV-uninfected population are represented for alcohol [54], diet [50, 51], sex, and anal receptive intercourse [55]. **B** Sequencing data were collated from the American Gut Project and prior analyses of the AGEhIV cohort and were processed in identical fashion [46, 48, 52] using dada2 [56]. For both datasets, Canberra beta-diversity matrices were calculated, and PERMANOVA tests were performed to quantify significance and effect sizes of ecological distances between cases and controls for each disease. Sample sizes are shown in parentheses encompassing balanced cohorts of cases and controls matched for confounding variables displayed at top left. For HIV cohorts, PERMANOVA statistics were calculated on five total sample groups from two studies [46, 52] including the following: men who have sex with men (*n* = 76) [46], females (*n* = 38) [46], men who have sex with women (*n* = 34) [46], combined females and males (irrespective of sexual behavior (148) [46], and a separate cohort of men who have sex with men (*n* = 102) [52]

Other known confounding variables that influence the microbiome include age, body mass index (BMI), sex, alcohol intake, and certain dietary intake patterns [48]. PLWH can have unique alcohol intake distributions [49], suggesting this variable ought to be captured in future studies, and that intake should be adjusted for or matched between comparison groups to mitigate confounding effects. Studies in which dietary intake was measured have not found significant differences between PLWH and people without HIV [50, 51], though further exploration is warranted. However, even when PLWH and people without HIV are matched for the aforementioned microbiota-confounding variables such as in the AGEhIV cohort study, significant differences in gut microbiome composition have been observed [46, 52]. Due to widely varying methods of microbiome analysis (including differences in quality filtering, read processing, beta-diversity assessment, and statistical analyses), it can be difficult to compare the magnitude of microbiome compositional differences between cases and unaffected controls across studies. Hence, putting such microbiome differences in context of what is observed for other human diseases is challenging. Using the American Gut Project [48, 53], which encompasses individuals that self-reported 19 different diseases as well as healthy control subjects, we applied an identical analytical pipeline as that employed in the AGEhIV cohort studies [46, 52]. We found that HIV-associated dysbiosis was among the strongest disease-associated dysbioses, second to inflammatory bowel disease and stronger than the remaining 18 diseases (Fig. 1).

### Microbiome in cardiovascular diseases in the general population

It is beyond the scope of this review to give a detailed description of dysbiosis in separate cohorts of CVD in the general population, and for details, we refer to comprehensive reviews by us [57] and others [58]. In brief, most studies published from cohorts of coronary artery disease (CAD) and HF reported depletion of different bacterial genera or species from the Ruminococcaceae and Lachnospiraceae families, which are microbiome patterns that are also observed in PLWH cohorts adjusted for MSM status. Weaknesses of many of the early microbiota studies include limited sample size and lack of essential covariates like diet and clinical data. More recent and comprehensive studies like the MetaCardis cohort have shown a complex interplay between microbiome and metabolomics features of the cardiometabolic disease spectrum from acute coronary syndromes to chronic heart failure [59].

### HIV-related or comorbidity-related dysbiosis

Few studies in PLWH have evaluated the relationship of the gut microbiome and cardiovascular disease, and most of the published studies have so far focused on cardiovascular risk factors such as metabolic syndrome, or subclinical atherosclerosis measured in a research setting, including carotid artery plaques and research coronary angiography.

The COCOMO study follows > 1000 PLWH for > 10 years for comorbidities, with available microbiome profiles in > 400 of these participants. Controls were recruited both from the general population and from a pre-exposure prophylaxis (PrEP) cohort of MSM. After separate comparisons of PLWH and controls in MSM and non-MSM strata, depletion of Lachnospiraceae and Ruminococcaceae and increase in Gammaproteobacteria and Desulfovibrionaceae were identified as HIV-related dysbiosis [60]. This HIV-related dysbiosis was associated with a doubled adjusted risk for the metabolic syndrome (MetS), mostly driven by increased risk of diabetes, hypertension, and abdominal obesity among the MetS components. Of note, there was an increasing association between dysbiosis index and MetS in PLWH with nadir CD4 T-cell counts less than 200, whereas in individuals who never developed immunodeficiency, the association was not evident. Furthermore, the HIV-related microbiome was associated with 30 cm^2^ larger area of visceral adipose tissue on abdominal CT scan, but again, only in those with previous severe immunodeficiency. This could possibly be a result of several factors including long-term viral replication, toxic ART, and a permanently damaged gut mucosa [60].

The AGEhIV study is another well-powered cohort study that has been collecting biological samples for over 10 years with a focus on comorbidities among individuals aged > 45 years and has found concordant results as those above. Namely, Lachnospiraceae and Ruminococcaceae were depleted in PLWH and Gammaproteobacteria and Desulfovibrionaceae were enriched in PLWH [46]. This study found that microbiota diversity was significantly lower in PLWH than controls, and that microbiota diversity was inversely correlated with circulating soluble urokinase plasminogen activator receptor (suPAR) [46]. This marker has been associated with CVD incidence in both PLWH [61, 62] and the general population [63, 64] and may contribute to CVD via activation and recruitment of monocytes [65]. Additionally, work from the AGEhIV cohort found that HIV-associated dysbiosis was significantly greater in PLWH that went on to develop CVD as compared to matched PLWH that did not experience CVD [46]. Associations between nadir CD4 count and HIV-associated dysbiosis were evident in this cohort [46], as it was in the COCOMO cohort and other studies [66, 67].

The largest study of gut microbiota and manifest atherosclerosis in PLWH to date included 361 women in the USA that were assessed by ultrasonography for the presence of carotid artery plaques. The study identified enrichment of *Fusobacterium* and *Proteus* and depletion of *Odoribacter* and *Adlercreutzia* in women with plaque compared to women without plaque. These bacteria correlated with plasma lipids, which were associated with increased risk of incident carotid artery plaque during 7 years of follow-up. Of note, this was not HIV specific, as the same associations were observed in women without HIV [68]. A smaller study found no significant composition differences between PLWH with and without coronary heart disease (CHD), despite lower alpha diversity in participants with CHD [69].

In a recent work from the COCOMO cohort, we found that PLWH with obstructive CAD assessed by CT angiography had clear shifts in their gut microbiota, with lower alpha diversity, increased beta diversity, compositional shifts including depletion of several bacteria from the Lachnospiraceae and Ruminococcaceae families, and increased relative abundance of *Ruminococcus gnavus*, a pro-inflammatory microbe associated with inflammatory bowel disease, as well as *Veillonella*. Of note, we identified no overlapping genera between CAD-related dysbiosis and the previously established HIV-related dysbiosis index, and the HIV-related dysbiosis index was not related to obstructive CAD (Trøseid et al., JID *in press*).

Whereas much of literature has focused on depletion of Lachnospiraceae and Ruminococcaceae families, enrichment of both *Ruminococcus gnavus* and *Veillonella* identified in the COCOMO cohort and *Fusobacterium* identified in the US cohort [68], have been identified in human carotid plaque studies [70], pointing to a potential causative or contributing role in the atherosclerotic process independent of HIV status. In light of the published studies in the field, it has so far not been possible to identify a clear gut dysbiosis associated with cardiometabolic comorbidities in PLWH across different cohorts. Moreover, the large inter-individual variation in gut microbiota composition has so far made it difficult to apply microbiota signatures as biomarkers for individual risk assessment.

## Microbial translocation

### Microbial translocation and HIV pathogenesis

A shared feature of the dysbiosis in cohorts of PLWH and cohorts of persons with CVD is the reduced potential for production of short-chain fatty acids (SCFA), including butyrate. Loss of butyrate-producing bacteria may result in a dysfunctional gut mucosal barrier, allowing passive leakage of microbial toxins such as LPS that binds to toll-like receptors and other receptors of the innate immune system, thereby triggering inflammation. This process is called microbial translocation and has been studied in several cohorts of PLWH, since first described by Brenchley et al. in 2006 [71].

### Microbial translocation and HIV comorbidities

In the general population, an increased potential for LPS biosynthesis in the microbiome has been reported among patients with CAD [72], and previous studies have linked circulating levels of LPS to insulin resistance [73], glycemic control and abdominal obesity [74], and cardiovascular events (reviewed in [75]). Atherosclerosis is in part an inflammatory process, and several lines of evidence suggest that LPS contributes to this process by fueling a low-grade chronic inflammation and atherothrombosis [75].

In PLWH, we and others have shown that circulating levels of LPS associate with several cardiovascular risk factors, including hypertension [76], insulin resistance, Framingham risk score [77], platelet reactivity [78], metabolic syndrome, central obesity, and hypertriglyceridemia [79]. The latter is possibly due to co-transportation with triglycerides in chylomicrons over the intestinal wall [80]. Despite these associations, circulating levels of LPS have not been linked to incident cardiovascular disease in PLWH. This could partly be due to low sample size in published studies and technical difficulties measuring LPS in bioassays, but it could also reflect that different forms of LPS have different biological properties.

Emerging evidence demonstrates different bioactivity of LPS, where hexa-acylated LPS triggers inflammation, while penta-acylated LPS does not [81]. One report from a CAD cohort showed that genes required for synthesis of the LPS O-antigen were enriched in CAD, whereas the lipid A module was depleted, probably due to depletion of *Bacteroides*, which produce non-inflammatory penta-acylated lipid A [82].

In a previously published probiotic trial including PLWH, we showed that gut bacteria producing hexa-acylated LPS were outnumbered by bacteria-producing penta-acylated LPS by a factor of 25, and that PLWH with a high ratio of hexa- to penta-acylated LPS-producing bacteria exhibited increased levels of systemic inflammation and tryptophan catabolism. Of note, changes in circulating LPS correlated closely to altered abundance of gram-negative bacteria producing penta-acylated LPS, including *Bacteroides* [83]. Hence, circulating LPS could partly reflect LPS from commensal microbes with low pro-inflammatory potential.

Indirect ways of measuring microbial translocation include measuring markers of immune cell responses to LPS, including circulating levels of soluble CD14 (sCD14) and LPS-binding protein (LBP) which are shed from toll-like receptor 4 upon LPS activation. Both markers have been associated with future cardiovascular events both in the general population [84] and in PLWH [85, 86]. However, both CD14 and LBP are promiscuous molecules with several triggers beyond LPS, and they should be regarded as markers of monocyte activation (sCD14) and general inflammation (LBP) rather than microbial translocation. Also, intestinal fatty acid-binding protein (IFABP) and zonulin are frequently reported in this context but should be regarded as markers of impaired gut barrier function rather than microbial translocation.

More specific quantification of microbes is possible via amplification and sequencing of microbial nucleic acids via 16S rRNA amplicon sequencing or via amplification-free sequencing of total nucleic acids. However, application of these techniques to low biomass samples such as blood and internal organs has been a challenge [87, 88] due to the risk of low-level environmental contaminants dominating results [87–91] and the difficulty in unambiguous distinguishment of such contaminants from the true signal. Such contaminants can come from tubes, tools, the skin of the study participant (as skin must be broken for a needle to collect blood), the skin from study staff, reagents, sample cross-contamination during DNA extraction and amplification, and index hopping during sequencing. This may explain mixed results among studies examining 16S rRNA in the blood of PLWH [92, 93]. Whereas most microbiota studies have focused on the bacteriome, the much less studied fungiome has also been reported to be altered in a few studies including PLWH [94] and could translocate to circulation and trigger inflammation [95]. Interestingly, a study reported that plasma β-d-glucan, a marker of fungal translocation, was higher in PLWH with carotid artery plaque compared to those without plaques [96].

Overall, it has so far remained challenging to measure microbial translocation, making it difficult to assess its potential role as a biomarker for HIV-associated comorbidities and to assess efficacy of experimental therapies that target this mechanism.

## Microbial metabolites and cardiovascular risk

Whereas microbiota traits vary from individual to individual and are affected by several confounding factors, including sexual practice and medicines, circulating metabolites may be less variable and therefore easier to evaluate as biomarkers. The microbiome is a complex bioreactor that produces and catabolizes neurotransmitters, amino acids, short-chain fatty acids, lipids, vitamins, and metabolites [97]. Several of these have been linked to different noncommunicable diseases in the general population, but not always to underlying gut microbiota dysbiosis. This section will focus on potential circulating biomarkers associated with CVD and underlying dysbiosis, with discussion of important aspects to consider when applying such biomarkers in PLWH.

### Short-chain fatty acids (SCFA)

SCFA are key gut microbial metabolites derived from fiber fermentation that benefit numerous facets of host biology. They are the primary energy source for the epithelial cells that line the colon, they induce tight junction proteins that bolster integrity of the gut epithelial barrier, and they induce regulatory T cells that dampen exuberant inflammation [98–100]. All of these functions may be protective in both HIV and CVD, making microbiome-mediated SCFA potentially important in pathology of both of these two disease states.

While over a third of microbial proteins have unknown function [101], many enzymes involved in the production of SCFAs have been identified. For this reason, abundance of these can be quantified in human stool via high-throughput sequencing of microbial DNA (metagenomics) or microbial RNA (metatranscriptomics). Decreased metagenome-encoded potential for SCFA production has been observed across microbiota studies examining individuals with CVD or PLWH [102, 103], suggesting a depletion of bioavailable SCFA is characteristic of these conditions.

Measuring SCFA directly in humans is stymied by several factors. It is estimated that 95% of SCFA produced by gut microbes are absorbed by the time fecal material reaches the rectum [104, 105]. Epithelial transmembrane transporters that are responsible for SCFA uptake into host tissues are upregulated with increasing exposure to SCFA in a dose-dependent fashion [52, 106], suggesting that high SCFA production can be matched by high uptake. For example, an observation of low SCFA in stool could either be the result of high SCFA production having been matched by high expression of SCFA transporters and high SCFA uptake, or of low SCFA production along with low SCFA transport expression and uptake. Thus, the remaining SCFA in feces may be a poor surrogate for microbiome-mediated SCFA production. Indeed, mixed results have been observed when examining SCFA levels in stool of PLWH and controls [107–111]. Murine studies examining microbiome-mediated SCFA production predominantly quantify SCFA in cecal contents [100, 112, 113], which anatomically precede the uptake that occurs in the colon. However, it is not feasible to collect human luminal material at the ileocecal junction, making SCFA quantification in humans challenging. We and others have found that measuring SCFA in serum, the compartment that may represent the SCFA pool post-uptake from the gut lumen, yields biologically meaningful results that are consistent with metagenome-encoded SCFA production capacity [52, 114]. Indeed, serum levels of the SCFA propionate in a cohort of PLWH correlated more strongly with metagenome-encoded abundance of propionate metabolism enzymes than did levels of propionate in stool [52].

Butyrate production from gut microbes may be particularly challenging to quantify in vivo because it is rapidly taken up by epithelial cells which then rapidly consume it, for butyrate is the preferred energy source for colonic epithelial cells. Propionate, on the other hand, is not the preferred energy source [115] and may thus be exported to the serum more so than butyrate. We found that abundance of butyrate-producing enzymes in the microbiome was not correlated with either stool nor serum levels of butyrate [52], highlighting the difficulty of measuring in vivo butyrate production from the gut microbiome.

### SCFA in PLWH

Several HIV microbiome studies have found a depletion of SCFA-producing gut bacteria in PLWH compared to controls [116, 117]. Studies have also found lower relative abundance of genes involved in SCFA production within metagenomes of PLWH [102, 103]. In a cohort of matched PLWH and controls, we have recently found that serum levels of the SCFA propionate were significantly reduced in PLWH, and that the conversion of lactate, one of several precursors for SCFA, was associated with CVD in PLWH [52]. While abundance of butyrate-producing enzymes in the microbiome was dramatically reduced in PLWH, we did not find differences in either circulating or stool butyrate levels, possibly because of the aforementioned biological fate of butyrate produced by the microbiome.

### SCFA in CVD

Diets rich in fiber, the primary substrate for microbial SCFA production, are promoted as being among the principal effective interventions [118, 119] to reduce blood pressure, a major contributor to CVD [120, 121]. These dietary recommendations are supported by the observed efficacy of dietary fiber intervention trials performed in the general (HIV-seronegative) population for reducing hyptertension [122, 123]. While other aspects of high-fiber diet may contribute to their protective role in CVD, murine studies demonstrate that SCFA alone can lower hypertension and CVD in animal models [124–126]. Mechanisms for these cardioprotective effects include induction of inflammation-dampening regulatory T cells [125], which reduce activation of various immune cells linked with CVD progression including macrophages. Another putative mechanism for the effects of SCFA on hypertension includes direct regulation of blood pressure in the kidneys via renal olfactory receptors [124]. Finally, as discussed above, SCFA strengthen gut barrier integrity and help mitigate microbial translocation, which itself may spur CVD in both PLWH and the HIV-negative population.

### Carnitine metabolites

The most compelling evidence of a link between the gut microbiome and CVD has been related to microbial metabolism of the dietary factors phosphatidylcholine and L-carnitine to trimethylamine-N-oxide (TMAO). The source of TMAO is TMA which is produced by the gut microbiota from nutrients containing L-carnitine or phosphatidylcholine and subsequently oxidized in the liver by flavin-containing monooxygenases to TMAO [127]. In particular, carnitine is abundant in red meat; hence, TMAO and other carnitine metabolites are potential links between dietary factors, gut microbiota, and CVD. In a landmark paper from the Hazen group [128], TMAO was identified as a strong predictor of CAD, and subsequent studies have linked TMAO to other types of CVD including acute coronary syndrome and chronic HF [129–132]. TMAO has been mechanistically linked to thromboembolic events as it enhances thrombus formation [133]. Furthermore, precursors of TMAO promote foam cell formation and atherosclerosis in animal models, but not when adding antibiotics to the drinking water, suggesting a microbiota dependent mechanism [134]. However, a firm link to disease-specific dysbiosis has not been convincingly demonstrated [57].

TMAO has been assessed in several cohorts of PLWH with conflicting results. Some studies have found an association with CVD [135], others did not [136], and one study showed a U-shaped association between TMAO and CVD in PLWH [137]. In a prospective cohort of 520 PLWH in the USA, plasma TMAO was associated with increased risk of incident carotid artery plaque, independent of traditional and HIV-related risk factors, during a median follow-up of 7 years, although the association was attenuated after further adjustment for markers of monocyte activation [135]. In contrast, in a longitudinal nested case–control study of first-time MI in PLWH from Denmark, we found no evidence for increased TMAO levels across several time points before onset of MI. However, TMAO levels increased significantly after initiation of ART, in particular in those starting a protease inhibitor-containing regimen [136]. Hence, we speculate that certain drugs including ART could interfere with microbial generation of TMA or with hepatic oxidation from TMA to TMAO, making TMAO a less suitable biomarker in PLWH.

Interestingly, a separate work reported that TMA was associated with carotid atherosclerosis in PLWH [138]. However, TMA is more volatile than TMAO, making it difficult to measure. Furthermore, another study showed that one of the TMAO precursors choline, but not TMAO, was associated with progression of carotid atherosclerosis in PLWH [135]. Other TMAO precursors such as trimethyl lysine (TML) have been associated with atherosclerosis in the general population [139–141], but to the best of our knowledge, not in PLWH. However, TMAO precursors such as carnitine, choline, and TML are probably more diet-related than microbiota-related metabolites and will in most likely have a limited role in advancing our understanding the contribution of the gut microbiota on comorbidities in PLWH.

### Uremic toxins

The role of microbiota-derived uremic toxins could be of particular relevance for cardiovascular risk in relation to chronic kidney disease (reviewed in [142]). Emerging evidence suggests that one such uremic toxin, phenylacetylglutamine (PAGln), which accumulates in children with urea cycle disorders, provides prognostic information on cardiovascular risk in association with chronic kidney disease [143] and even in populations without renal failure [144]. Similar to TMAO, PAGln is mainly a bacterial degradation product, which is derived from phenylalanine-rich food and undergoes subsequent conjugation with glutamine in the liver [144]. In the general population, PAGln has been associated with the risk of ischemic stroke and atrial fibrillation [145] and recently also with coronary CAD [146] and HF [147].

PAGln signals within host cells via G protein-coupled receptors, including adrenergic receptors [148].The link between PAGln and CVD was first established by an untargeted metabolomics approach, demonstrating that the gut microbiome contributes to circulating levels of PAGln, and that PAGIn could enhance platelet adhesion and thrombus formation [148].

So far, there are limited data on PAGln in PLWH. Similar to TMAO, levels of PAGln were reported to increase in PLWH treated with ART, with higher levels in PLWH with hyperglycemia and/or hyperlipidemia [149]. Studies of PAGln in relation to cardiovascular comorbidities in PLWH are yet to be performed but should take into account potential impact of ART and renal dysfunction in the study design.

### Secondary bile acids

Whereas bile acids are traditionally regarded as emulsifiers to facilitate the absorption of dietary fat and fat-soluble vitamins, bile acids are also recognized as signaling molecules that interact with plasma membranes as well as nuclear receptors, exerting regulatory effects on energy homeostasis [150], lipid and glucose metabolism [151], and other physiological processes [152]. In the gut, primary bile acids undergo metabolism to *secondary* bile acids, before reabsorption as a part of the enterohepatic cycle (reviewed in [153]). These microbial bile acid modifications have major impact on the agonist activity on the bile acid receptors such as the farnesoid X receptor which has several pleiotropic effects [154] and could represent a link between the gut microbiome and CVD.

We have previously analyzed the circulating bile acid pool in patients with HF and healthy controls and found an increased ratio of secondary to primary bile acids in HF which was associated with reduced overall survival in unadjusted, but not in adjusted analyses [155]. Bile acids are technically difficult to measure. With the exception of a study reporting higher levels of primary and secondary bile acids, as well as microbiome alterations in PLWH with chronic HCV infection and a history of major depression [156], data on circulating bile acid pool is so far limited in PLWH.

### Tryptophan metabolites of the kynurenine pathway

Kynurenine pathway metabolites can be produced via the catabolism of tryptophan by the host enzyme indoleamine 2,3-dioxygenase 1 (IDO1), which is induced in the setting of inflammation. This enzymatic pathway serves to limit T-cell proliferation via tryptophan starvation and by the direct action of kynurenine compounds (e.g., kynurenine, 3-hydroxyanthranilic acid) on T cells [157] including the induction of regulatory T cells. Kynurenine compounds also diminish differentiation of Th17 cells, which are critical mediators of gut barrier integrity and are characteristically depleted in the gut of PLWH that initiated treatment during the chronic phase [158]. This gut Th17 cell depletion is associated with elevated markers of inflammation and possibly microbial translocation [158]. Serum kynurenine/tryptophan (KT) ratio, a surrogate marker for activity of the kynurenine metabolic pathway, is in turn associated with mortality and Th17 cell depletion in PLWH [159, 160]. While IDO1 is induced by inflammatory cytokines and is expressed highly in the gut of PLWH with progressive infection [160], its expression is diminished in the treated PLWH despite persistently elevated KT ratios in this subject group [44]. We previously found that gut-resident microbes encode enzymes with analogous functions to that of IDO1, and that the abundance of gut bacteria that encoded such enzymes correlated with KT ratios in treated PLWH, while gut IDO1 expression itself did not [44]. Fecal metabolomics have concordantly found kynurenine metabolites elevated in PLWH [161], further suggesting that microbes may contribute to the immunomodulatory kynurenine pathway of tryptophan catabolism in PLWH.

In the general population, several studies have linked increased KT ratio to increased risk of diabetes and CAD [162, 163]. In PLWH, several studies have reported the kynurenine pathway to associate with mortality [159, 164, 165], non-AIDS comorbidities, aging, and inflammation, and the kynurenine pathway has been suggested to be of particular importance in connecting gut inflammation with age-related comorbidities [30]. Studies in PLWH have linked tryptophan metabolism to gut microbiota alterations and different aspects of atherosclerosis, including endothelial dysfunction [166] and carotid atherosclerosis [167–169], although the links between dysbiosis, tryptophan catabolism, and cardiovascular disease have been incompletely defined. In the COCOMO cohort, we found that increased KT ratio mediates around 10% of the association between gut microbiota alterations and visceral adipose tissue accumulation [170], suggesting this metabolic pathway may also be linked with adiposity. Some of the strongest associations between mortality and KT ratios are evident in sub-Saharan African populations [159, 164, 165]. Although tryptophan levels have been consistently lower in several cohorts of PLWH [30], tryptophan levels were in general markedly lower in a sub-Saharan cohort than those reported for developed countries, suggesting that lower tryptophan intake related to malnutrition could be of importance in addition to the inflammatory-induced tryptophan depletion [171]. Given the primary source of bioavailable tryptophan is dietary, the interplay between diet and microbiota in influencing kynurenine pathway activity and its links to these important adverse biological phenomena merits further exploration.

### Microbiota-derived indoles

In addition to kynurenines, tryptophan can also be metabolized into serotonin (5-hydroxytryptamine) as well as into indole and its derivatives, the latter through the gut microbiota-dependent indole pathway [172]. Indole and its derivatives have been linked to protective (e.g., indole-3-propionic acid; IPA) and detrimental (e.g., indoxyl sulfate) effects on inflammation and vascular disease. Whereas indole and IPA are important for the gut mucosal barrier function in part by exerting anti-inflammatory activities through activation of aryl hydrocarbon receptor and pregnane X receptors, indoxyl sulfate has cardiotoxic and nephrotoxic properties [173, 174]. Indoxyl sulfate is associated with cardiovascular risk related to chronic kidney disease [174], and as it accumulates with decreased renal clearance, it is also considered a microbiota-derived uremic toxin, as discussed above.

A recent study of women with and without HIV evaluating a broad range of tryptophan metabolites along the kynurenine and indole pathway found that plasma levels of IPA and IPA/kynurenic acid ratio were inversely associated with carotid artery plaque, regardless of HIV serostatus [175]. Of note, five gut bacterial genera and many affiliated species were positively associated with IPA, including *Roseburia* sp., *Eubacterium* sp., *Lachnospira* sp., and *Coprobacter* sp*.*, whereas no bacterial genera were found to be associated with kynurenic acid, suggesting a beneficial role of IPA and its bacterial sources in atherosclerosis and CVD [175].

### The histidine metabolite imidazole propionate

Imidazole propionate (ImP) is a microbially produced histidine metabolite. ImP has been linked to insulin resistance and type 2 diabetes through the mammalian target of rapamycin complex (mTORC) pathway [176, 177] and was recently reported to provide prognostic information and to be related to dysbiosis in patients with HF [178, 179].

ImP production has been linked to certain bacteria, including *Ruminococcus gnavus* and *Veillonella* [176]. As we found in the COCOMO cohort these bacteria to be related to obstructive CAD in PLWH, we hypothesized that circulating ImP levels could be a potential biomarker of obstructive CAD. We found elevated ImP levels to be associated with both obstructive CAD and the underlying dysbiosis [180]. However, whereas dysbiosis index was independently associated with obstructive CAD, the association with ImP was attenuated and no longer significant in multivariable analyses.

Our findings are in line with a recent report of ImP being associated with carotid atherosclerosis and underlying dysbiosis in women living with HIV [181]. Further analysis identified additional bacterial species and bacterial hutH gene (encoding enzyme histidine ammonia-lyase in ImP production) associated with plasma ImP levels, and that a gut microbiota score based on these ImP-associated species was positively associated with plaque and several pro-inflammatory markers [181]. Hence, ImP seems to capture cardiovascular comorbidities and underlying dysbiosis in PLWH irrespective of gender or mode of transmission. So far, no studies have reported ImP in relation to CAD in the general population, but, in light of our findings, such studies are warranted.

## Integrating dysbiosis and microbial metabolites

### Unbiased versus targeted omics approach

With the combined genes of the microbiome approaching that of the total human genome, and each microbe having the potential to turn on and off the production of hundreds of metabolites, several undiscovered microbiota-related metabolites are likely to be relevant for HIV-associated comorbidities. Whereas most studies to date have been based on sequencing the 16S rRNA gene, metagenomic sequencing presents an opportunity to better define functional changes in the gut microbiome. Ultimately, combined analyses of the actual byproducts of microbial activity (unbiased metabolomics and/or proteomics analyses of parallel plasma samples) and microbiota (16S rRNA or metagenomics) analyses controlling for relevant confounders may augment discovery [57, 181]. Furthermore, the gut virome and mycobiome are underexplored areas in PLWH, and both may impact immune function [182, 183].

A recent study applying unbiased multi-omics approach on the COCOMO cohort identified separate clusters of PLWH with different metabolic risk profiles. Although analyses were adjusted for confounders including sexual preference, the high-risk cluster was partly driven by a *Prevotella*-enriched gut microbiota with a high proportion of MSM, and there was little overlap between microbiota profiles and plasma metabolomics and lipidomics, respecitively [184]. In contrast, a recent study of PLWH discovered ImP as the most promising of several soluble markers through an unbiased multi-omics approach and also found a clear correlation between ImP and dysbiosis as well as carotid atheroslcerosis [181]. Of note, the latter study only included women living with HIV; hence, the confounding effect of MSM was not relevant.

There is a risk that unsupervised, unbiased multi-omics approach in data set with a large proportion of MSM will not be able to filter out the MSM signal without a clear strategy, preferably by including seronegative control groups of MSM and non-MSM, either as part of the study cohort or by applying data from data repositories. An alternative is to establish an HIV-associated dysbiosis index in a data set with relevant control groups or to establish a comorbidity-related dysbiosis index if the MSM proportion is equal in those with and without comorbidities.

A more targeted approach is needed to confirm or reject promising findings from unbiased discovery studies. The choice of candidate biomarkers can be made by different strategies, either based on specific hypotheses or by certain traits in gut dysbiosis pointing to specific biomarkers to be tested, i.e., depletion of Ruminococcaceae and Lachnospiraceae in relation to circulating SCFAs [52], alteration of tryptophan metabolizing bacteria in relation to KT-ratio [44] or IPA levels [175], or increase in *Ruminococcus gnavus* in relation to ImP levels [180].

## From biomarkers to clinical application

### Risk stratification beyond traditional risk factors

For translation to a clinical setting, biomarkers that are easily measurable in a reproducible way in plasma, urine or other body fluids, will probably be easier to implement than individual microbiota signatures, given the complexity and variability of the latter. A clinically relevant microbiota-related biomarker should preferably be associated with the disease-related dysbiosis or other microbiota traits, as well as with the comorbidity in question, independent of relevant covariates. For HIV-related comorbidities, this should include traditional and HIV-related risk factors, as well as potential confounders, such as mode of transmission, antibiotics, other relevant drugs, and dietary data.

However, for use in a clinical setting, a novel biomarker should also provide additional information beyond established biomarkers or at least independent of established biomarkers. In non-HIV cohorts, TMAO has been shown to provide information on risk of major cardiovascular events after myocardial infarction independent of troponin levels and in independent cohorts [129] but is yet to be established as a risk marker in an acute clinical setting [130].

In PLWH, a major advance was recently published on screening for precursors of anal cancer by measuring microbial proteins from anal swabs [185]. Of note, microbiota differences were limited and driven by outliers, whereas microbiota-derived proteomics separated clearly and converged on common pathways related to energy metabolism. Of note, measurements of two downstream substances, cobalamin and succinyl-CoA, in two independent cohort from Madrid and Milano, were able to increase sensitivity and specificity dramatically compared to anal cytology [185]. These data need to be reproduced in independent studies before affecting screening algorithms for anal cancer in PLWH, and similar requirements should be made for emerging biomarkers for other comorbidities.

### Therapeutic target

Another potential clinical application of the gut microbiota is as a therapeutic target. With respect to comorbidities, a microbiota-directed intervention should preferably demonstrate improvement of the comorbidity in question or its risk factors. Several attempts have been made to target the microbiota with probiotics (live beneficial bacteria), prebiotics (food for beneficial bacteria), or synbiotics (probiotics combined with prebiotics) in PLWH. In a comprehensive review summarizing these trials, there is no evidence that any of these interventions are clinically helpful in PLWH [186]. Although several trials have reported effect on one or two biomarkers, typically a cytokine or a subset of immune cells, these biomarkers differ between trials, the primary end point is often not clearly defined, and most trials have been underpowered [186].

Other attempts have been made to use fecal microbiota transplant (FMT) with different target population and donor selection strategies. Although FMT appears safe, there is limited evidence for better immunological recovery or efficacy in modulating other established, clinically relevant outcomes in PLWH [187, 188]. Of note, engraftment was transient and limited after one-time transplant [189] and even with repeated weekly inoculations regardless of antibiotic pre-treatment [188], underscoring the challenges of inducing lasting changes in gut microbiota composition via FMT.

Alternative approaches for therapeutic strategies include attempts to target the enzymatic pathways of bacterial metabolites, such as production of TMAO or ImP, in a “drug the bug” strategy [190]. So far, identifying suitable biomarkers will be required before choosing more targeted approaches in PLWH.

### Tool for precision medicine

It was recently shown in the REPRIEVE trial that pitavastatin, a cholesterol-lowering drug, reduced the relative risk of a major cardiovascular event by around one third in PLWH at low to moderate risk of cardiovascular disease [191]. As the risk reduction occurred irrespective of baseline LDL cholesterol levels, other mechanisms such as anti-inflammatory effects of statins could be relevant. Of note, there is at present no single biomarker to guide selection of candidates for statin therapy, beyond global risk assessment.

The tremendous inter-individual variation in gut microbiota composition is clearly a limitation in biomarker studies, but this variation could potentially be used as a tool for precision medicine. An elegant study showing substantial inter-individual variation of glycemic responses after different meals also showed that integration of microbiota profiles and metadata in a machine learning model made it possible to precisely predict individual glycemic responses in order to personalize nutritional advice [192]. Whether such an approach could be used to tailor individualized prophylaxis, including statin therapy to prevent HIV-related comorbidities is promising but yet unknown. Indeed, the gut microbiome can metabolize therapeutic drugs [193], and the gut microbiomes across individuals exhibit substantial heterogeneity in their capacity to metabolize different drugs [194]. Studies to test the prediction of optimal individualized therapies would require real-time integration of microbiota profiles (or related metabolites), drugs, traditional risk factors, and HIV-specific factors to assess, i.e., 10-year risk of myocardial infarction or stroke. Such a prediction tool should be able to outperform established risk score algorithms, which generally underestimate CVD risk in PLWH, and be fairly easy to use in a clinical setting. Nevertheless, with rapid advances in artificial intelligence, such an approach could be feasible in the near future.

## Future research

As shown in Table 1, several metabolites demonstrated to be of relevance in the general population are understudied in PLWH. This includes among others circulating bile acids, uremic toxins and partly histidine metabolites, and carnitine metabolites beyond TMAO. On the other hand, studies performed in PLWH should also inspire studies in the general population, such as circulating propionate levels, which should be investigated as a potential dysbiosis-related cardiovascular biomarker in HIV and non-HIV cohorts.

**Table 1:** Candidate microbiota-related biomarkers in HIV and non-HIV cohorts

**Biomarker**	Relevance and main findings	Limitations	Future directions
Carnitines, including TMAO	• Predicts clinical end points in numerous studies in the general population [57] • Reproducible measurements • Conflicting or negative results in PLWH [136–138]	• TMAO levels influenced by diet, renal and liver function, and potentially ART interfering with hepatic oxidation • Circulating TMAO weakly linked to dysbiosis [57]	• Microbiota-derived TMAO precursors such as TML [141] should be studied in PLWH • Potential therapeutic target in pharmacological products interfering with TMA production [190]
Short-chain fatty acids	• Low SCFA production linked to dysbiosis in CAD/HF [57] • Also linked to dysbiosis in PLWH when adjusted for MSM status [46, 60] • Low circulating propionate in PLWH [52]	• Measurable in snap frozen fecal samples without preservatives but rapidly degraded • Low circulating levels of butyrate, not suitable as soluble biomarker	• Circulating propionate should be investigated as a microbiota-related CVD biomarker in PLWH and the general population • Potential therapeutic target in high fiber dietary interventions
Markers of microbial translocation	• Increase in LPS-producing microbes linked to dysbiosis in non-HIV cohorts of CVD [57, 75] • Increased plasma LPS linked to cardiovascular risk factors in PLWH [76–79] • Increased LBP linked to CAD in HIV cohort [86]	• Direct measurement of gut permeability is so far not feasible in the clinic • Large variability in LPS LAL assay • LAL assay does not separate between hexa- and penta-acylated LPS variants [81, 83]	• Need of better standardization of LPS measurements • Other markers of bacterial translocation such as LBP, I-FABP, beta-glucan, and zonulin should be further studied
Tryptophan catabolism through kynurenine and indole pathways	• KTR predicts CVD in the general population [162, 163] • In PLWH, KTR is linked to dysbiosis [44], mortality, and non-AIDS comorbidities [165–170], and IPA is negatively associated with carotid plaques [175]	• Limited association with gut microbiota [170], affected by systemic inflammation and protein intake [171]	• Tryptophan-derived kynurenines and indoles should be investigated in large prospective cohorts for incident CVD in PLWH
Bile acids	• Increased primary to secondary bile acid ratio in HF [155] and in HIV/HCV coinfected [156] • Otherwise little data in PLWH	• Large variability and technically difficult to measure	• Circulating bile acid pool and dysbiosis should be investigated in PLWH • Pleiotropic effects of bile acid receptor FXR [154] should be further studied
Uremic toxins, including PAGIn	• Microbiota-generated toxins accumulate with reduced urinary excretion [142] • PAGIn predicts CVD in the general population [146, 147]	• Renal function is a major confounder, ART potential confounder • Intervention with sevelamer targeting uremic toxins negative in PLWH [195]	• PAGIn should be investigated in PLWH
Histidine metabolites, including ImP	• ImP related to type 2 diabetes [176] and heart failure [178, 179] • Related to dysbiosis, obstructive CAD, and carotid atherosclerosis in PLWH [180, 181]	• So far, only cross-sectional studies in PLWH and in the general population • Mechanism of action not clarified	• ImP should be investigated in large prospective cohorts for incident CVD in PLWH and in the general population

*CAD* coronary artery disease, *HF* heart failure, *CKD* chronic kidney disease, *KTR* kynurenine/tryptophan ratio, *LPS* lipopolysaccharide, *LBP* LPS-binding protein, *LAL-assay* limulus amebocyte lysate assay, *I-FABP* intestinal fatty acid binding protein, *ImP* imidazole propionate, *IPA* indole-3-propionate, *FXR* farnesoid X receptor, *PAGIn* phenylacetylglutamine, *TMA* trimethylamine, *TMAO* trimethylamine-N-oxide, *TML* trimethyllysine

Furthermore, gut microbiota analysis beyond the bacteriome should be applied to precisely define the HIV-related mycobiome and virome and further assess the cross-kingdom microbiome in relation to comorbidities. Moreover, with the rapid advances in artificial intelligence, it is of importance to make clear strategies on how to overcome specific confounding factors, such as sexual preference, when planning unbiased multi-omics analyses.

Finally, once promising gut microbiota-related candidate markers have been identified in various studies, such markers should be independently validated in adequately powered multicenter prospective cohorts designed to assess biomarkers in relation to incident comorbidities, such as the MISTRAL study which is nested in the EuroSIDA cohort. Such studies should ultimately lay the foundation for future precision medicine, including novel strategies for personalized risk assessment and intervention studies targeting the gut microbiota to reduce the risk of HIV-related comorbidities.

## Data Availability

No datasets were generated or analysed during the current study.
